# Pre-scan state anxiety is associated with greater right amygdala-hippocampal response to fearful versus happy faces among trait-anxious Latina girls

**DOI:** 10.1186/s12888-023-05403-6

**Published:** 2024-01-02

**Authors:** Dana E. Díaz, Wan-Ling Tseng, Kalina J. Michalska

**Affiliations:** 1 Department of Psychology, University of California, Riverside, CA USA; 2 Department of Psychiatry, Columbia University Irving Medical Center, New York, NY USA; 3 Yale Child Study Center, Yale School of Medicine, Yale University, New Haven, CT USA

**Keywords:** State anxiety, Trait anxiety, Amygdala-hippocampus, Inferior parietal lobe (IPL), Latina youth, functional magnetic resonance imaging (fMRI)

## Abstract

**Background:**

Unfamiliarity with academic research may contribute to higher levels of anticipatory state anxiety about affective neuroimaging tasks. Children with high trait anxiety display differences in brain response to fearful facial affect compared to non-anxious youth, but little is known about the influence of state anxiety on this association. Because reduced engagement in scientific research and greater mistrust among minoritized groups may lead to systematic differences in pre-scan state anxiety, it is crucial to understand the neural correlates of state anxiety during emotion processing so as to disambiguate sources of individual differences.

**Methods:**

The present study probed the interactive effects of pre-scan state anxiety, trait anxiety, and emotional valence (fearful vs. happy faces) on neural activation during implicit emotion processing in a community sample of 46 preadolescent Latina girls (8–13 years).

**Results:**

Among girls with mean and high levels of trait anxiety, pre-scan state anxiety was associated with greater right amygdala-hippocampal and left inferior parietal lobe response to fearful faces relative to happy faces.

**Conclusions:**

Anticipatory state anxiety in the scanning context may cause children with moderate and high trait anxiety to be hypervigilant to threats, further compounding the effects of trait anxiety. Neuroimaging researchers should control for state anxiety so that systematic differences in brain activation resulting from MRI apprehension are not misleadingly attributed to demographic or environmental characteristics.

**Supplementary Information:**

The online version contains supplementary material available at 10.1186/s12888-023-05403-6.

## Introduction

Accurate recognition of others’ emotional expressions provides us with cues to salient environmental features and the emotional state of our interaction partners. Children with anxiety exhibit threat biases in processing and interpreting facial affect [1–4] and display atypical patterns of neural activation when viewing threat stimuli [5]. Perceiving social cues as threatening may cause anxious children to avoid social situations, reducing opportunities to habituate or reappraise fears and further exacerbating anxiety symptoms [6]. Momentary feelings of anxiety in healthy children, or *state anxiety*, can also elicit behavioral and neural responses to negative emotional stimuli resembling those seen in trait anxiety and anxiety disorders [7–10]. Many children experience elevated state anxiety while undergoing functional magnetic resonance imaging (fMRI) scanning [11]. However, few developmental studies on anxiety test how pre-scan state anxiety influences neural substrates of emotion processing, so it is unknown whether previous findings capture trait features of anxiety or state features associated with apprehension of the neuroimaging environment or both.

Given that some demographic groups may experience greater state anxiety about scanning due to limited experience with research or medical mistrust [12–14], it is critical to understand the neural correlates of elevated state anxiety during emotion processing, and whether they are distinct from, or overlapping with those seen in trait anxiety. In the present study, a community sample of Latina girls (8–13 years) completed an fMRI implicit emotion processing task, during which they viewed fearful and happy faces varying in emotion intensity and reported the face’s gender. We tested the interactive effects of state anxiety, trait anxiety, and emotional valence on girls’ neural responses to facial affect.

Anxious children display increased threat vigilance to fearful and angry facial affect relative to non-anxious children [1, 3, 4, 15, 16] and are more likely to appraise emotional stimuli as negative or threatening [2, 17–19]. Several brain networks are associated with threat biases in children and adults with anxiety disorders. Children and adults with anxiety disorders display elevated activation in the amygdala and insula to negative or ambiguous facial affect [20–29], atypical recruitment of prefrontal, executive control networks [30–34], and reduced connectivity between these networks [35–41]. The amygdala and insula are highly interconnected structures that contribute to the peripheral expression of emotion and salience detection [42–44]. Upon viewing a threat, the amygdala signals the production of a threat response and increases vigilance [45]. The insula is involved in interoception and plays a crucial role in subjective emotions [46, 47]. Given these regions’ central role in identifying a stimulus’ emotional significance and generating an affective response [48, 49], they are hypothesized to be important for processing negative and positive affective information [50]. Thus, amygdala and insula hyperactivity may contribute to anxiety symptoms, such that negative social information is assigned greater salience in anxious than in non-anxious individuals.

The MRI context itself can elicit temporary feelings of state anxiety, discomfort, or even panic among children [11, 51], which may evoke patterns of activation that are unique from [52], or overlapping with, trait anxiety and anxiety disorders [53]. State anxiety in the fMRI context is an essential consideration in pediatric anxiety research, but, to date, it is understudied (see Michalska et al. (2020) [54] for a detailed review of methodological considerations and challenges of the scanning environment). Children undergoing fMRI scans are often alone in the confined space in the scanner bore, where they must tolerate loud noises and restricted motion [55]. This experience can elicit physical discomfort [56] and anxiety [11, 51] and increase biological indices of stress like cortisol [57, 58]. Elevated state anxiety can impact task performance [59, 60] and influence attentional [61, 62], perceptual [63–66], and interpretative mechanisms [59, 67, 68]. State anxiety also elicits changes in blood oxygen level-dependent (BOLD) response while participants view threatening or emotional stimuli [7–10] and even during rest [52, 69]. Importantly, although undergoing MRI scanning induces stress in about 30% of participants [11], few studies test how pre-scan state anxiety impacts subsequent task performance. Further, although state anxiety can interact with trait anxiety to predict behavioral responses to negative emotional stimuli [60, 70, 71], little is known about how state and trait anxiety interact to predict brain activation during affective neuroimaging tasks.

Undergoing an MRI scan is anxiety-inducing, not just for children [51, 72], but also more generally for people unfamiliar with the scanning environment [73, 74]. Because minoritized groups like Latinx participants are underrepresented in research and, for historic reasons, display greater mistrust in medical, academic, and scientific institutions than white participants [12–14], there may be systematic differences in state anxiety across demographic groups that lead to inaccurate interpretations of results. For instance, higher rates of pre-scan state anxiety among a minoritized group in a study may lead to greater alterations in emotion processing, cognitive functioning, or physiology in that group [75]. Without accounting for state anxiety, such task-related differences could be misattributed to temperamental, environmental, or cultural factors rather than apprehension of the research environment.

In the present study, a community sample of predominantly Mexican American girls (8–13 years) completed an implicit face emotion viewing task while in an MRI scanner. During this task, children viewed graded levels of happy and fearful faces varying in emotion intensity and reported on the face’s gender. We tested the interactive effects of trait anxiety and state anxiety on mean neural response to emotional facial affect. To evaluate whether interactive effects of state and trait anxiety were specific to threatening facial affect, we also tested whether emotional valence (fearful vs. happy) moderated any observed associations. We hypothesized that both state and trait anxiety would be associated with increased amygdala and insula response to fearful facial affect [20, 21, 23, 76]. Following prior work showing that state anxiety increases attentional and interpretive threat biases only for trait-anxious people [60, 70], we also hypothesized that state and trait anxiety would interact to predict amygdala and insula response to fearful affect, such that state anxiety would increase activation among trait-anxious youth. As preliminary work observes trending associations between childhood anxiety and amygdala response to positive social cues [77, 78], we did not have specific predictions about the effects of state or trait anxiety on happy facial affect. Instead, we hypothesized that the effects of anxiety on activation in salience processing regions would be greater for fearful than happy facial affect.

## Methods

### Participants and procedure

Fifty-five 8-13-year-old Latina girls and their primary caregivers were recruited from the Inland Empire of Southern California to participate in a longitudinal study of emotional development. Participants were recruited via the Psychology Department’s shared database of child participants recruited from the community. Participant eligibility was determined by phone screening with the primary caregiver. Children were eligible for participation if they were between 8 and 13 years old, proficient in English, right-handed, and had no contraindications for neuroimaging (e.g., no ferrous metal in the body, not pregnant, not claustrophobic). Children also needed to be at least 50% Latinx origin and self-identify as Latina to be eligible for participation (see Table 1 for ethnic-racial identity of participants in the final sample). Exclusionary criteria included a current psychiatric diagnosis of Tourette’s syndrome, obsessive-compulsive disorder, lifetime history of mania, psychosis, or pervasive developmental disorder. Menstruation onset was initially used as an exclusionary criterion but was dropped to increase sample size, and two postmenarchal participants were recruited.

**Table 1:** Racial and ethnic background of study participants (*N* = 46)

Ethnic Background	*N*
Latina	39
Mexican	31
South/Central American	2
Mixed ethnicity (Mexican & other Latinx)	6
Mixed race (Latina & white)	7

Participants completed a laboratory testing session and a scanning session. During the laboratory session, children and caregivers reported on family demographics and children’s behavior, anxiety, and other mental health outcome measures not reported here. During the scanning session, children completed an implicit face emotion viewing task while undergoing fMRI data collection. fMRI scans were not collected from seven participants because they did not return for the scan visit (*n* = 4), or due to participants’ distress (*n* = 1), dental braces (*n* = 1), or experimenter error (*n* = 1). Two participants were excluded due to low response rate on the task (> 25% missed trials), resulting in a final sample of 46 participants (*M*_*age*_ = 9.9 ± 1.2 years; Table 2) and their caregivers (40 mothers, 6 fathers). The visit structure was changed part-way through data collection, so 11 participants completed the laboratory session and scanning session at two visits, approximately two weeks apart (*M* = 19.7 days, + 4.8). The remaining 35 participants completed the laboratory and scanning sessions in one visit. Results were largely unchanged when days elapsed between visits was added as a covariate (Table S1). Upon participant arrival at each wave, written parent consent and child assent were obtained. At the end of each session, participants were compensated with a gift card and a toy. All study procedures were approved by the Institutional Review Board. All data were collected prior to the COVID-19 pandemic.

**Table 2:** Sample demographic characteristics and descriptive statistics for study variables

Characteristic	Descriptive Statistics
*N*	46
Female (%)	100
Age, years	
Mean (SD)	9.9 (1.2)
Range	8–12
Household income (*N* = 45)	
Mean (SD)	$60,666 ($46,277)
Range	<$5,000 - >$180,000
STAIC-Trait	
Mean (SD)	38.4 (7.2)
Median	39
Range	22–54
Skew	-0.17
STAIC-State (*N* = 43)	
Mean (SD)	29.5 (5.3)
Median	30
Range	20–48
Skew	0.41
SCARED	
Mean (SD)	36.8 (14.6)
Median	35
Range	4–71
Skew	0.46

STAIC = State-Trait Anxiety Inventory for Children. SCARED = Screen for Child Anxiety Related Disorders. Household income was not available for one participant. STAIC-State measures were not collected from three participants

### Measures

#### Demographic characteristics

Psychological research is commonly conducted among white, educated, upper- and middle-class samples [79]. Thus, communities with lower income or less access to education may have less exposure to, and more apprehension of, scientific research. We tested whether state anxiety was associated with individual differences in household income, parental education, and children’s perceived social standing relative to their community and the United States. As our sample was fairly homogenous in ethnicity and city of residence, we did not test for associations of state anxiety with these variables.

Parents indicated their child’s age and ethnic background, as well as their own educational background and household income. We also measured children’s self-reported subjective socioeconomic status using a modified version of the MacArthur Scale of Subjective Social Status [80]. This two-question measure probed individuals’ perceptions of where they rank in the status hierarchy of (1) their community, and (2) the United States. Participants ranked their status by indicating the rung on a nine-rung ladder, on which the top of the ladder represented “people who are the best off, those who have the most money, most education, and best jobs” and the bottom represented “people who are the worst off, those who have the least money, least education, worst jobs, or no job.”

#### State and trait anxiety symptoms

**Fig. 1:**
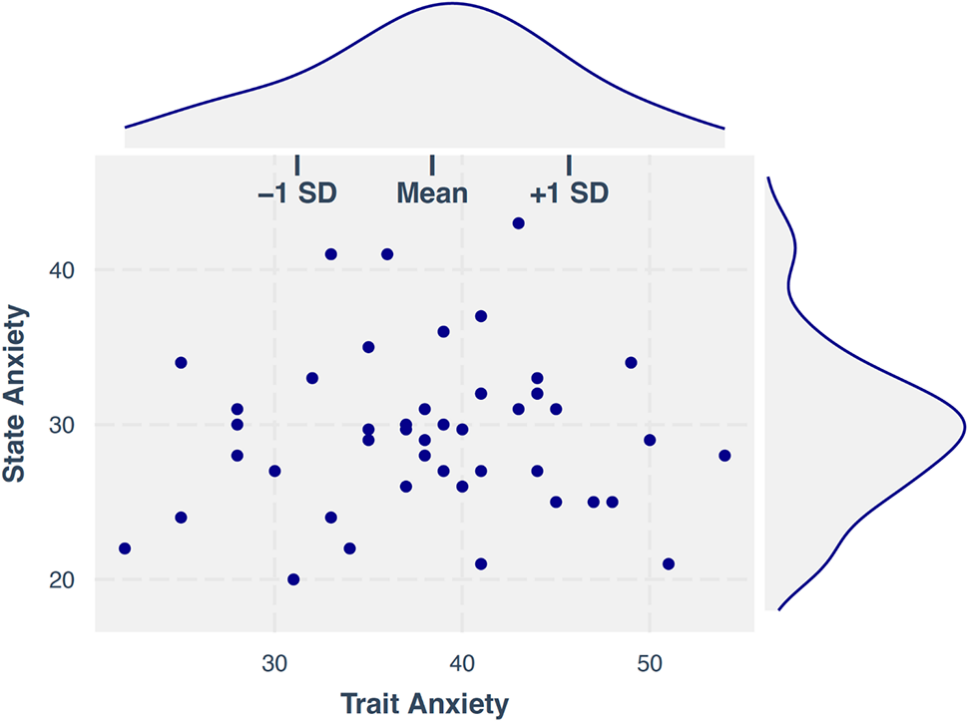
Scatterplot and density distributions for participants’ state and trait anxiety. *Note*: State and trait anxiety were assessed via the State-Trait Anxiety Inventory for Children (Spielberger et al., 1973); SD = standard deviation

Children’s state and trait anxiety symptoms were measured via child self-report on the State-Trait Anxiety Inventory for Children (STAIC) [81] (Fig. 1). The STAIC is comprised of two 20-item scales that assess state anxiety (STAIC-State) and trait anxiety (STAIC-Trait). Children respond to all items on a three-point Likert scale, and each subscale is summed to a total score (range: 20–60). Both measures display excellent internal consistency (*α* ≥ 0.81) [82]. The STAIC-State measures state anxiety by asking children to indicate how they feel “right now…at this moment.” State anxiety was measured in the imaging facility immediately before the scan. The STAIC-State was added to the protocol shortly after data collection began, so three participants did not complete it and their scores were imputed with mean-replacement. The STAIC-Trait assesses trait levels of anxiety by probing how the child usually feels. Children’s trait anxiety was measured in the lab, prior to the scan. The STAIC-Trait demonstrates concurrent validity with other anxiety measures (*r* = .88) [82]. In our sample, the STAIC-Trait was highly correlated with child report on the Screen for Child Anxiety Related Disorders, *r* = .81, *p* < .001 (SCARED) [83], which assesses anxiety disorder symptomatology. State and trait anxiety scores on the STAIC were uncorrelated in our sample, *r* = .04, *p* = .77.

Although our sample was a non-treatment-seeking community sample, children’s self-reported anxiety scores were notably elevated, according to their self-report on the SCARED (Table 2). The mean score was 38.6, and more than 80% of participants surpassed the threshold for clinically significant anxiety levels (≥ 25; *N* = 38). These scores are substantially higher than those self-reported by clinically anxious girls and boys (7–18 years; *M*_SCARED_ = 23.8) [84] and female psychiatric outpatients (6–17 years; *M*_SCARED_ = 25.8) [85].

As mentioned above, due to the change in visit structure mid-way through data collection, 11 participants reported trait and state anxiety at separate visits, approximately two weeks apart (*M* = 19.7 days, ± 4.8). Post hoc sensitivity analyses were conducted to include the time elapsed between state and trait anxiety collection, which had minor influences on reported effects (see Supplement).

#### Implicit face emotion viewing task

**Fig. 2:**
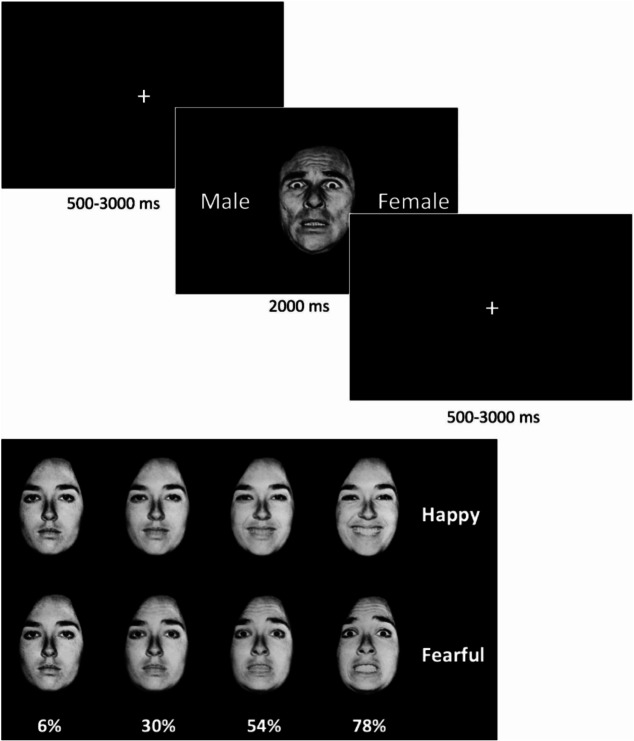
Implicit emotion viewing paradigm with happy and fearful facial stimuli. Participants viewed black and white faces that were morphed blends between neutral and fearful or happy emotional expressions at 6%, 30%, 54%, and 78% emotion intensities. Participants responded with the gender of the face. Faces were presented in random order for 2000 ms each, followed by a 500-3000 ms jittered interstimulus interval

While undergoing fMRI scanning, children completed an implicit face emotion viewing task [86, 87], during which they labeled the gender of ten actors’ face emotion pictures (100% white; 60% female). Faces were morphed between neutral and fearful or happy expressions at 6%, 30%, 54%, and 78% emotion intensity (Fig. 2). Faces were presented in random order for 2000 ms each, followed by a 500–3000 ms jittered interstimulus interval (ISI) during which a white fixation cross was presented against a black background. In one run, children viewed 20 trials of each morphed fearful and happy stimulus, summing to 160 trials total and 80 trials of fear morphs. The task was programmed in E-prime (version 2.0.10; PST Inc., Pittsburgh, PA). Participants viewed the back-projected screen via a mirror mounted on the head coil and pressed a button box with their right hand to indicate the gender of the face (male/female).

### Imaging data

#### MRI data acquisition and preprocessing

Whole-brain neuroimaging data were collected using a 3T Siemens Prisma scanner and 32-channel head coil. Two hundred and forty functional image volumes were collected during one 9 min 44 s run. Functional image volumes with 62 contiguous interleaved axial slices were obtained with a T2*-weighted echo-planar sequence (TR = 2500 ms; TE = 32 ms; flip angle = 80; Field of View [FOV] = 204 × 228 mm; matrix = 102 × 114; voxel size = 2 × 2 × 2 mm^3^). Using a magnetization-prepared gradient echo sequence, functional data were anatomically localized and coregistered to a high-resolution T1-weighted volumetric scan of the whole brain that was collected prior to the functional volumes (MPRAGE: TR = 2400 ms; TE = 2.72 ms; TI = 1060 ms; flip angle = 8; FOV = 240 × 256 mm; matrix = 300 × 320; voxel size = 0.8 x 0.8 x 0.8 mm^3^).

Individual echo-planar images were preprocessed and analyzed using AFNI (Analysis of Functional NeuroImages; version 22.0; Cox, 1996 [88]). Preprocessing included despiking, slicetime correction, motion correction, and smoothing with a 4 mm full-width at half-maximum (FWHM) kernel. All MRI data were transformed to Montreal Neurological Institute (MNI) space. BOLD data was scaled at the voxel-wise time series by their temporal means so effect estimates can be interpreted as percent signal change. Every TR on which motion exceeded 2 mm was censored, and excessive motion was defined as more than 20% of TRs censored for motion/outliers (*N* = 0). One participant had 20.4% of TRs censored and was included in all analyses. Average head motion was not correlated with state anxiety (*r* = .15, *p* = .30) or trait anxiety (*r* = .24, *p* = .10). Using the AFNI 3dDeconvolve function, a general linear model was generated to estimate mean task-related activation for happy and fearful facial affect, averaged across all emotion intensities. Third-order Legendre polynomials modeled baseline drift and six head motion parameters.

### Statistical analyses

#### Behavioral analyses

Task performance was assessed via gender labeling accuracy, percent of trials participants responded to, and mean reaction time. Children’s state and trait anxiety levels on the STAIC were correlated with each behavioral measure.

Correlations were also tested between state anxiety and household income, parental education, and children’s perceived social standing relative to their community and the United States. Bonferroni correction was conducted to correct for multiple comparisons across the four demographic measures at *p* < .0125.

### Neuroimaging analyses

To correct for multiple comparisons, familywise error correction was performed using Monte Carlo simulation on gray matter-masked, whole-brain data (3dClustSim in AFNI). The gray matter mask was created by segmenting the MNI152_2009c anatomical template into gray matter and non-gray matter. Masked output maps included gray matter voxels of the whole brain. The voxel threshold of *p* < .005 resulted in an average cluster threshold of 50 voxels at the whole-brain corrected alpha level of 0.05. Peak coordinates (x, y, z) are reported based on the MNI atlas in left, posterior, inferior (LPI) orientation.

**Fig. 3:**
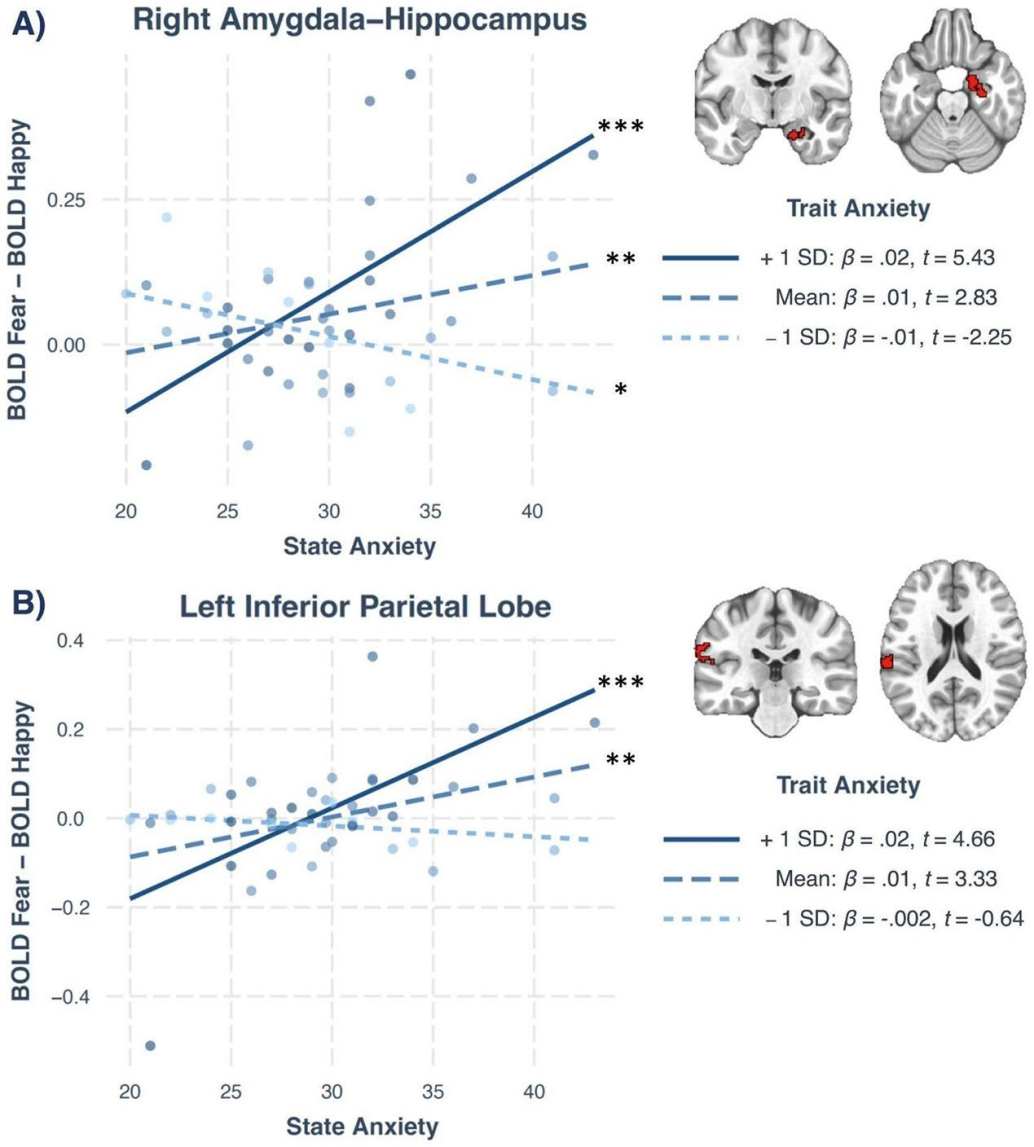
Trait anxiety moderated associations between state anxiety and neural responses to fearful versus happy faces. Results of the gray matter-masked, whole-brain linear mixed effects model. Trait anxiety moderated associations between state anxiety and differential neural responses to fearful versus happy faces (BOLD_Fear_ – BOLD_Happy_) in the (A) right amygdala-hippocampal complex, and (B) left inferior parietal lobe. Simple slopes depict the association between state anxiety and mean neural activation for children with low (-1 SD; STAIC-Trait = 31.2), mean (STAIC-Trait = 38.4), or high (+ 1 SD; STAIC-Trait = 45.7) trait anxiety. STAIC = State-Trait Anxiety Inventory for Children. Groups displaying slopes that significantly differ from 0 are indicated on the legend: ***p ≤ .001, **p ≤ .01, *p ≤ .05

A linear mixed-effects model was conducted using AFNI’s 3dLME program [89]. The model tested the independent and interactive effects of state anxiety, trait anxiety, and emotional valence in predicting mean task-related activation to fearful and happy faces, averaged across all intensity levels. Gray matter-masked, whole-brain voxel-wise tests were used for all fMRI analyses. Age and motion were included as covariates of no interest. Given our focus on neural responses to fear, in clusters with significant three-way interactions, we subtracted the mean neural response to happy facial affect from the mean neural response to fearful facial affect to capture BOLD response to fearful relative to happy facial affect. State anxiety was plotted on the x-axis, difference in average brain activation (BOLD_Fear_ - BOLD_Happy_) was plotted on the y-axis (Fig. 3). Follow-up simple slopes analyses tested the model-predicted slope for children with high (+ 1 SD), mean, and low (-1 SD) trait anxiety levels. In clusters with significant State Anxiety x Trait Anxiety interactions, average brain activation was calculated, collapsed across fearful and happy facial affect. Follow-up simple slopes analyses tested the model-predicted slope for the association between state anxiety and mean neural activation for children with high (+ 1 SD), moderate (mean), and low (-1 SD) trait anxiety levels.

## Results

### Behavior

Two participants responded to fewer than 75% of trials and were excluded from the final analyses with *N* = 46. Overall, children showed high task engagement (*M* = 94.6% response rate ± 5.2%) and were accurate at labeling the gender of faces (*M* = 91.2% ± 8.2%). State anxiety was inversely associated with average response rate across all fear trials, *r* = − .31, *p* = .039, and happy trials, *r* = − .32, *p* = .028, such that participants with greater state anxiety responded to fewer trials. Trait anxiety was not associated with response rate for fear or happy trials (*ps* > 0.27). Gender labeling accuracy was not correlated with state anxiety or trait anxiety for fear or happy trials (*ps* > 0.06). Average response time (*M* = 1075.0 ms ± 120.6 ms) was also unrelated to state anxiety and trait anxiety (*ps* > 0.50).

Post hoc linear regression analyses were also conducted to examine whether state and trait anxiety interacted to predict behavioral outcomes, controlling for age. State and trait anxiety did not interact to predict accuracy, average response time, or response rate (all *ps* > 0.21).

### Effects of demographic characteristics on pre-scan state anxiety

Children’s pre-scan state anxiety was inversely correlated with the community subscale of the MacArthur Scale of Subjective Social Status, *r* = − .33, *p* = .023. In other words, children who rated themselves as lower in social standing relative to their community tended to have greater state anxiety prior to the MRI scan. However, this result did not hold after Bonferroni correction. Pre-scan state anxiety was not associated with how children rated themselves relative to people in the United States, *r* = − .16, *p* = .30. Children’s pre-scan state anxiety was also not associated with objective demographic variables, including parental education or household income, *ps >* 0.48.

### Brain activation

#### State Anxiety x Trait Anxiety x Emotional Valence

State and trait anxiety interacted with stimuli’s emotional valence to predict mean BOLD response in two clusters. The first cluster encompassed portions of the right amygdala and hippocampus (*k* = 107, x = 27, y = -15, z = -23), and the second cluster was in the left inferior parietal lobe (IPL: *k* = 90, x = -57, y = -21, z = 35). Differential neural responses to fearful versus happy faces were calculated by subtracting mean neural responses to happy expressions from mean responses to fearful expressions, within each significant cluster. Follow-up simple slopes were conducted in each cluster to test the model-predicted association between state anxiety and differential neural response to fearful versus happy faces (BOLD_Fear_ - BOLD_Happy_) for children at low (-1 SD: 31.2), mean (38.4), and high levels of trait anxiety (+ 1 SD: 45.7).

In the right amygdala-hippocampal complex, simple slopes revealed a positive association between state anxiety and differential neural responses to fearful versus happy faces (BOLD_Fear_ - BOLD_Happy_) for children with mean, *β* = 0.01, *SE* = 0.002, *t* = 2.83, *p* = .007, and high levels of trait anxiety (+ 1 SD), *β* = 0.02, *SE* = 0.004, *t* = 5.43, *p* < .001 (Fig. 3a). In other words, with increasing state anxiety, children with mean and high trait anxiety displayed greater responses to fearful relative to happy facial expressions in the amygdala-hippocampal complex. By contrast, children with low trait anxiety (-1 SD) displayed an inverse association between state anxiety and differential neural response (BOLD_Fear_ - BOLD_Happy_), *β* = − 0.01, *SE* = 0.003, *t* = -2.25, *p* = .030, such that state anxiety was associated with decreased neural responses to fearful relative to happy facial affect.

In the left IPL, simple slopes revealed a positive association between state anxiety and differential neural responses to fearful versus happy facial affect (BOLD_Fear_ - BOLD_Happy_) for children with mean, *β* = 0.01, *SE* = 0.003, *t* = 3.33, *p* = .002, and high levels of trait anxiety (+ 1 SD), *β* = 0.02, *SE* = 0.004, *t* = 4.66, *p* < .001 (Fig. 3b). As with the right amygdala-hippocampal complex, with increasing state anxiety, children with mean and high trait anxiety displayed greater left IPL responses to fearful relative to happy facial affect in left IPL. No such associations emerged for children with low trait anxiety (-1 SD), *β* = − 0.002, *SE* = 0.004, *t* = -0.64, *p* = .53.

### Independent and interactive effects of state and trait anxiety

A two-way interaction emerged between state and trait anxiety in predicting mean BOLD response in the right caudate (*k* = 90, x = 19, y = 19, z = 7), averaged across fearful and happy facial affect. Simple slopes tested the model-predicted association between state anxiety and mean BOLD response to all emotional affect for children with low (-1 SD: 31.2), mean (38.4), and high trait anxiety (+ 1 SD: 45.7). We observed an inverse association between state anxiety and mean neural response to emotional affect for children with mean, *β* = − 0.02, *t* = -3.12, *p* = .003, and high trait anxiety (+ 1 SD), *β* = − 0.04, *t* = -5.14, *p* < .001. State anxiety was not associated with mean right caudate activation at low levels of trait anxiety (- 1 SD), *β* = 0.01, *t* = 1.47, *p* = .15.

We also observed main effects of state anxiety, trait anxiety, and emotional valence. State anxiety was inversely associated with right caudate response to emotional affect (*k* = 141, x = 11, y = 19, z = -3). Trait anxiety was inversely associated with activation in the right inferior temporal gyrus (*k* = 76, x = 53, y = -25, z = -23). There was also a main effect of emotional valence in a cluster spanning portions of the right amygdala and hippocampus (*k* = 89, x = 23, y = -5, z = -23), such that the cluster was more reactive to fearful than happy faces.

### Multivariate outlier detection

A *post hoc* multivariate outlier detection analysis was performed to identify outliers. The Mahalanobis distance was calculated using participants’ state anxiety scores, trait anxiety scores, and mean activation to fearful and happy facial affect within each significant cluster. No participants had a Mahalanobis distance exceeding the χ^2^(14) critical of 39.25 at *p* < .001, and all data was retained.

## Discussion

Youth unfamiliar with academic and biobehavioral research settings may experience elevated levels of state anxiety in anticipation of affective neuroimaging tasks, complicating inferences. The present study probed the independent and interactive effects of state anxiety, trait anxiety, and emotional valence on neural activation during implicit emotion processing in a community sample of preadolescent Latina girls with elevated trait anxiety. State anxiety was associated with children’s subjective social status (prior to multiple comparison correction) but was uncorrelated with trait anxiety. Neuroimaging analyses revealed a three-way interaction between state anxiety, trait anxiety, and emotional valence in the right amygdala-hippocampal complex and left IPL. For children with mean and high levels of trait anxiety, state anxiety was associated with greater activation to fearful relative to happy facial affect in both clusters.

Although our sample was a non-treatment-seeking community sample, we observed high levels of self-reported trait anxiety. Over 80% of participants met criteria for clinically significant anxiety on the SCARED, underscoring the importance of community-informed anxiety research focused on Latinx youth [90]. This observation is in line with other research finding high rates of anxiety in Latinx youth [91–93], and Latina girls specifically [94], relative to other ethnic groups. Although this is an understudied issue, some research suggests that discriminatory experiences [95], acculturative stress [96], cultural factors [97], or parenting [98] may contribute to elevated anxiety.

Somewhat surprisingly, trait and state anxiety subscales on the STAIC were uncorrelated in our sample. State and trait anxiety are sometimes correlated in research settings [99] but not always [52]. This variability is likely because state anxiety measurements are closely tied to the specific context in which they are collected. Certain stimuli, such as scary movies, might induce anxiety in many children, while others might be more situation-specific, such as fear of flying, dentists, or anticipating a brain scan. Preliminary research suggests that state and trait anxiety have unique patterns of neural activation at rest [52], reinforcing the notion that they are distinct constructs [100] (though see [53], which finds induced anxiety parallels the effects of pathological anxiety in the insula and medial prefrontal cortex). Hence, these results suggest that the MRI setting may selectively elicit anxiety in certain youth. Moreover, other factors like unfamiliarity with the scanning environment [73, 74] and distrust of medical or scientific institutions [12–14] may contribute to systematic variations in state anxiety across demographic groups [101]. Psychological research is often conducted in white, educated, and affluent communities [79]. People with lower income and/or less access to education may have limited exposure to scientific research, contributing to discomfort or mistrust. Unfortunately, we did not collect measures that allowed us to directly test whether discomfort in or mistrust of the research environment specifically contributed to greater state anxiety. However, our study revealed an inverse association between state anxiety and subjective social status (prior to multiple comparison correction), such that children who rated their family as having a lower standing in the community tended to have higher pre-scan state anxiety. State anxiety was not associated with objective measures of socioeconomic status (e.g., household income, parental education), nor with children’s subjective assessment of their family’s status compared to the broader United States. These findings suggest that the scanning environment may be particularly anxiety-inducing for participants who feel marginalized relative to other members of their community. It is important to note that our study exclusively involved Latina girls residing in the Inland Empire, resulting in a relatively homogenous sample. Future research should validate this hypothesis among participants representing diverse socioeconomic, educational, and ethnic backgrounds, and directly probe participants’ experience with and trust in neuroimaging or research settings.

Neuroimaging analyses revealed that state and trait anxiety interacted with emotional valence to predict average neural activity in a cluster encompassing portions of the right amygdala-hippocampal complex. Our amygdala hypotheses were partially confirmed, such that for moderately and highly trait-anxious children, state anxiety was associated with greater right amygdala-hippocampal activity for fearful faces, compared to happy faces. The amygdala and hippocampus are highly interconnected limbic structures that are involved in processing emotional stimuli [102–104]. The amygdala is responsible for bottom-up, automatic threat detection and emotional arousal [29, 105–107]. The hippocampus facilitates emotion recognition and interpretation by contextualizing sensory input within emotional memories [102, 103, 108]. Thus, elevated amygdala-hippocampal activity can reflect increased vigilance or emotional response to threat stimuli. Both state and trait anxiety have been independently linked to elevated activation in the amygdala [7, 8, 22, 23] and hippocampus [109–111] in response to threatening facial affect. However, to our knowledge, this is the first study to find that trait anxiety moderates the association between state anxiety and amygdala-hippocampal activation to fear relative to happy facial affect. These results indicate that, among youth with moderate and high trait anxiety, state anxiety can exacerbate amygdala-hippocampal response to threat stimuli. Thus, fear or discomfort in MRI scanners can mimic or augment the effects of trait anxiety on amygdala-hippocampal activity, even among youth with mean levels of trait anxiety. During threat processing, the amygdala can also modulate brain activity in other brain regions, including perceptual [112, 113] and executive control networks [114–116]. Thus, amygdala hyperactivation associated with state anxiety may have widespread effects on brain function. Such compounding effects of trait and state anxiety have implications for investigating processes associated with clinical anxiety. For instance, moderately trait anxious youth categorized as “healthy controls” may exhibit elevated anticipatory state anxiety, causing them to resemble youth with high trait anxiety or clinical anxiety, thereby minimizing or obscuring differences between groups.

Surprisingly, for children with low trait anxiety, state anxiety was inversely associated with right amygdala-hippocampal activity for fearful compared to happy facial affect. In other words, low trait-anxious children with high state anxiety displayed greater right amygdala-hippocampal activity to happy faces, compared to fearful faces. This pattern differs from prior research finding state-anxious participants show greater amygdala response to fearful expressions [7, 117] and weaker amygdala reactivity to happy expressions [8]. Although our speculation is limited, it’s worth highlighting several potentially significant factors that could contribute to this result. Although better known for its role in threat processing, the amygdala also plays a role in reward processing and positive affect [50, 118], and therefore its activity may be elicited by happy faces. Certain personality traits are associated with individual differences in amygdala response to happy faces. For instance, extraversion, which is often higher in those with low trait anxiety [119], is associated with greater amygdala response to happy facial affect [120]. Thus, youth with low trait anxiety may display stronger amygdala-hippocampal responses to happy affect than their moderately and highly trait-anxious peers. However, in our sample, this pattern of activation was only observed in participants with both low trait anxiety and high state anxiety. Preliminary work finds that state anxiety can be associated with weaker amygdala response to fearful stimuli in specific contexts, such as following positive movies [121], suggesting that state-anxious participants might be more susceptible to the effects of positive emotional stimuli. Our data may therefore support a model whereby state anxiety can modulate participants’ susceptibility to both positive and negative biases; further testing would help confirm such a possibility.

Finally, we observed a three-way interaction between state anxiety, trait anxiety, and emotional valence in the left IPL. Again, for girls with mean and high levels of trait anxiety, state anxiety was positively associated with left IPL activity for fearful compared to happy facial affect. The IPL is involved in deliberate and sustained attention [122, 123], but elevated IPL activity during threat processing may also indicate hypervigilance [124]. Children with anxiety display hypervigilance to threats [1, 3, 15, 125–129] and right IPL hyperactivity during emotion processing [130]. Additionally, among anxious and non-anxious youth, negative affect is associated with left IPL-amygdala functional connectivity when appraising threat [131]. The pattern of activation in the IPL may therefore be related to parallel findings observed in the amygdala-hippocampal complex, though their contralateral location complicates this interpretation. Together, our results suggest that anticipatory anxiety in the scanning environment may cause children with moderate and high trait anxiety to be hypervigilant to threat, further compounding the effects of trait anxiety. These data align with prior work in adults showing that state anxiety is associated with hypervigilance to threat only for trait-anxious [70] and clinically anxious participants [132].

Several limitations of the current study and future research considerations should be acknowledged. First, our insula hypotheses were not confirmed, possibly because state and trait anxiety show overlapping patterns of neural activation in the insula [53], and thus, may not elicit interactive effects. Second, sample size was modest. Recent studies suggest that brain-behavior effects can sometimes be inflated and contribute to problems with replicability [133]. Further, examining interactive effects in modest samples may have limited our ability to detect significant effects, as a smaller proportion of the sample fell 1 SD above or below the mean on the STAIC-T. However, unlike some other methods to explore interactive effects, simple slopes analyses use the whole dataset to predict slopes at each level of the moderator, so these estimations were informed by the full sample. Despite the modest sample size, our study was strengthened by the fact that our sample consisted of Latina girls -- a demographic group that was under-represented in research. As mentioned previously, low representation of Latinx children in anxiety research is especially troubling given that they display high rates of anxiety [91–94, 97, 98]. Further, Latinx children are one of the largest and fastest-growing ethnic groups in the United States [134]. Thus, results may inform future large-scale studies by identifying preliminary effects within a well-characterized sample of Latina girls. Third, the cross-sectional study design limited our ability to make inferences about developmental processes. Future work should test longitudinal changes in the effects of state and trait anxiety on implicit fear processing, especially within executive control networks, which may display changing associations with anxiety across development. Fourth, because state anxiety measures were collected prior to the scan, we cannot be sure that such levels were sustained throughout the task. Thus, our results capture the effects of anticipatory, pre-scan anxiety on neural response during implicit emotion processing. A final limitation of this study is that all the faces presented in this experiment were non-Hispanic white people. All girls in this study identified as Latina and most were 100% Latina (~ 85%, *N* = 39), with the remainder of girls from both white and Latinx backgrounds (~ 15%, *N* = 7). Thus, for most participants, stimuli were from an outgroup (other) race-ethnicity. People respond differently to face stimuli that depict members of their own race compared with those of an outgroup race or ethnicity [135]. Outgroup members are more readily associated with aversive stimuli [136] and anxious arousal [137] than members of one’s own race or ethnicity. People also display differences in neural activation to racial ingroup versus outgroup faces [138, 139]. Thus, differences in participants’ experiences or familiarity with white people may have influenced their neural response to the face stimuli. Future work should sample faces from a variety of races and ethnicities and/or covary for participants’ experiences with racial outgroup members.

In summary, the present study examined the influences of trait anxiety and anticipatory, pre-scan state anxiety on Latina girls’ neural response to fearful and happy facial affect. Among girls with moderate and high levels of trait anxiety, state anxiety was associated with greater right amygdala-hippocampal and left IPL activity to fearful relative to happy facial affect. Together, these results suggest that anticipatory state anxiety in the scanning environment may cause children with moderate and high trait anxiety to be hypervigilant to threat, further compounding the effects of trait anxiety. Minoritized groups often have reduced engagement in scientific research and more mistrust [12–14], and thus may experience greater levels of pre-scan state anxiety. In the present study, girls who rated their family as having a lower community standing tended to have elevated pre-scan state anxiety (prior to multiple comparison correction), which may support that demographic factors like subjective social status influence children’s reaction to the research environment. Imaging researchers should survey and control for state anxiety so that any systematic differences in subgroups’ neural response resulting from MRI apprehension are not incorrectly attributed to demographic or environmental characteristics.

### Electronic supplementary material

Below is the link to the electronic supplementary material.

Supplementary Material 1

## Data Availability

The data in the current study are available from the corresponding author upon request.
