# Supplementary material for: Pre-scan state anxiety is associated with greater right amygdala-hippocampal response to fearful versus happy faces among trait-anxious Latina girls
Source: BMC Psychiatry. 2024 Jan 2;24:1. doi: 10.1186/s12888-023-05403-6 (PMC10759434; doi:10.1186/s12888-023-05403-6)
Supplement: Supplementary file 1 — Supplementary Material 1 [file 12888_2023_5403_MOESM1_ESM.docx]

**Supplementary Materials**

**Table S1. Results from a sensitivity analysis testing the influence of time elapsed between the collection of state and trait anxiety measures**

**
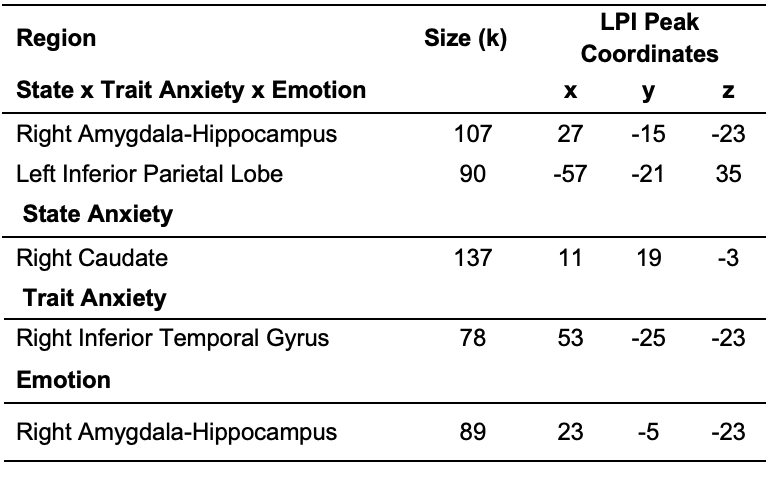
**

A sensitivity analysis tested the influence of variations in time elapsed between the collection of state and trait anxiety measures across participants. As in the primary analysis, a linear mixed-effects model tested the independent and interactive effects of state anxiety, trait anxiety, and emotion in predicting mean task-related activation, controlling for age and motion. Additionally, days elapsed between participant report on the STAIC state and trait anxiety measures was included as a covariate. Gray matter-masked, whole-brain voxel-wise tests were used.

**Effects of State and Trait Anxiety on High-Intensity Fearful and Happy Faces**

***Analysis***

To examine the effects of state and trait anxiety on high-intensity emotional faces, a general linear model was fit to each subjects’ preprocessed data (see MRI Data Acquisition and Preprocessing) using AFNI’S 3dDeconvolve and 3dREMLfit functions. Eight regressors were created, one for each emotion intensity (6% happy/fearful, 30% happy/fearful, 54% happy/fearful, and 78% happy/fearful) using the onset time for each trial in each condition, with fixation trials modeled as an implicit baseline. Third-order Legendre polynomials modeled baseline drift and six head motion parameters. Two linearly-weighted additional regressors were created to directly contrast high-intensity fearful and happy faces (78% happy/fearful) versus “neutral” faces (6% happy). The 6% happy face was selected as the “neutral” comparison for both regressors because it appears neutral to the eye (Figure 2) and enabled us to equate the baseline comparison across both models. In addition, anxious youth sometimes interpret neutral stimuli as negative or display biased brain responses to them [[140,141]](https://paperpile.com/c/Dh0eL7/4AROW+Denip), so slightly happy faces may reduce excessive negativity biases.

Linear mixed-effects models were conducted using AFNI’s 3dLME program [[88]](https://paperpile.com/c/Dh0eL7/mnEpH) to test the independent and interactive effects of state and trait anxiety on (1) fearful vs. neutral faces (78% fear vs. 6% happy) and (2) happy vs. neutral faces (78% happy vs. 6% happy). Both models included main and interactive effects of state and trait anxiety, controlling for age and head motion. Gray matter-masked, whole-brain voxel-wise tests were used for all analyses. Follow-up simple slopes analyses were conducted in clusters with significant state-by-trait anxiety interactions to obtain the model-predicted slope for children with high (+1 SD), moderate (0 SD), and low (-1 SD) trait anxiety levels.

***Results***

Trait anxiety was positively associated with brain response to fearful faces (vs. neutral, i.e., 6% happy) in the left insula (*k* = 59, x = -47, y = 3, z = 7) and right putamen (*k* = 60, x = 21, y = 9, z = 5). By contrast, trait anxiety was not associated with neural response to happy (vs. neutral) faces. We did not observe any main effects of state anxiety on neural response to fear or happy faces (vs. neutral). Finally, state and trait anxiety interacted to predict neural response to happy (vs. neutral) faces in the left precentral gyrus (*k* = 194, x = -57, y = -3, z = 33), left inferior frontal gyrus (*k* = 156, x = -37, y = 17, z = 27), and left fusiform gyrus (*k* = 57, x = -25, y = 3, z = -41). Follow up simple slopes analyses revealed that for low trait-anxious youth (-1 SD), state anxiety was positively associated with brain activation in all three regions, *β*s > .01, *p*s < .011. For high trait-anxious youth, state anxiety was inversely associated with activation in all three regions, *β*s < -.03, *ps* < .001. However, these effects were driven by two visual outliers and were no longer significant when they were removed. No significant associations emerged for youth with mean levels of trait anxiety. No State x Trait Anxiety interactions emerged for fearful faces (vs. neutral).
